# Fetal Brain Injury in Rhesus Isoimmunization Fetus

**DOI:** 10.7759/cureus.21450

**Published:** 2022-01-20

**Authors:** Eman Al Sanei, Mostafa Elbatreek, Rula B Sallout, Badi Al Baqawi, Bahauddin I Sallout

**Affiliations:** 1 Maternal Fetal Medicine, Women's Specialized Hospital, King Fahad Medical City, Riyadh, SAU; 2 Neonatal Intensive Care Unit, Children's Specialized Hospital, King Fahad Medical City, Riyadh, SAU; 3 Collage of Medicine, Al Faisal University, Riyadh, SAU

**Keywords:** hdrops, intrauterine transfusion, fetal anemia, encephalomalacia, ventriculomegaly

## Abstract

This report presents a rare fetal and neonatal complication brain injury (encephalomalacia and ventriculomegaly) as a consequence of severe fetal anemia resulting from Rhesus (Rh) isoimmunization. A 28-year-old gravida 4 para 3 woman was referred at 21+4 weeks of gestation to the fetal medicine clinic as a case of Rh isoimmunization. Fetal ultrasound showed a normal anatomy scan with normal brain structure, but with severe fetal anemia. The patient was treated with multiple intrauterine transfusions, but still developed complications post-transfusions.

This case shows that severe cerebral developmental anomalies can occur because of severe fetal anemia secondary to Rh isoimmunization, such as in this case - ventriculomegaly and encephalomalacia. It has been concluded that proper antenatal counseling and early intervention for severe fetal anemia are beneficial to prevent such complications from occurring. It is crucial to consider appropriate antenatal and postnatal radiological imaging for such cases.

## Introduction

ABO type, Rhesus (Rh{D}) status, and antibody screening should be determined in all pregnant women at the initial perinatal visit. The most common antigen causing isoimmunization is Rh(D). Only 17% of Rh(D)-negative women who do not receive prophylaxis immunoglobulin become immunized. Up to 90% cases of Rh isoimmunization can be prevented by routine postpartum immunoglobulin administration to Rh(D)-negative women, and >99% cases can be prevented if the immunoglobulin is given during the third trimester and postpartum.

## Case presentation

A 28-year-old gravida 4 para 3 woman with two living children presented with a medical history of rheumatic heart disease. The patient underwent aortic valve replacement with a tissue valve in 2012, during which she received many units of blood transfusion and developed Rh isoimmunization (blood group: A, Rh-negative and anti-D positive). Her first pregnancy was complicated by hydrops due to which she did not receive an intrauterine blood transfusion (IUT) and was delivered via cesarean section (C/S) prematurely. The second pregnancy was complicated by intrauterine fetal death and terminated before 24 weeks of gestation. The patient’s last pregnancy was managed with frequent IUTs, and she delivered a healthy newborn via cesarean section (C/S) at 36 weeks of gestation.

**Figure 1:**
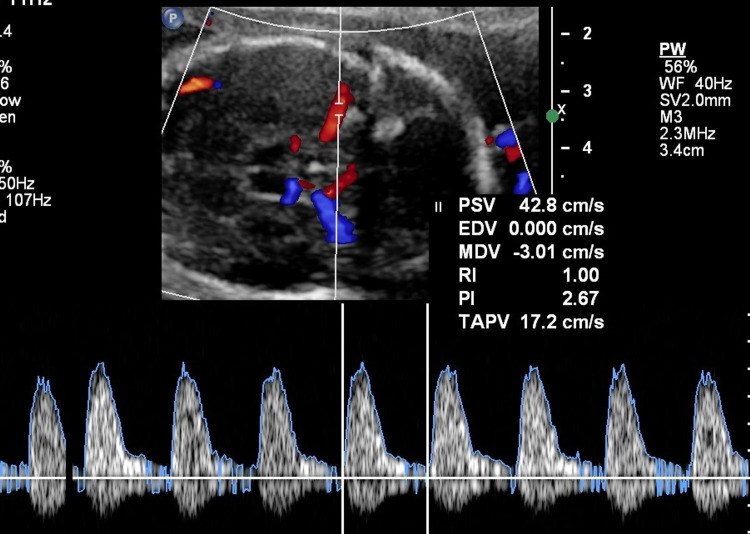
Doppler showing peak systolic velocity of 29.4 (1.60 MoM) MoM: multiple of median

**Figure 2:**
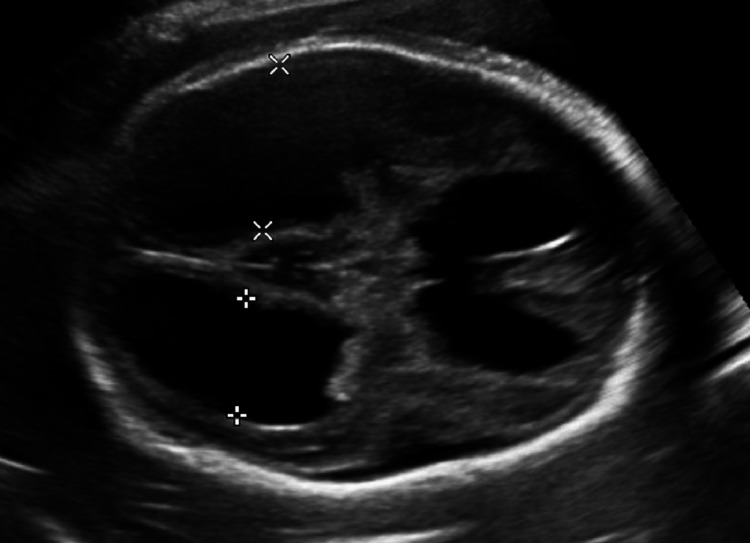
Ultrasound after the fourth transfusion showing severe ventriculomegaly (24 mm)

During the current pregnancy, the patient presented for the first time at 21 weeks and three days of gestation with an uneventful pregnancy. The anatomy scan revealed normal fetal anatomy without signs of hydrops but with middle cerebral artery-peak systolic velocity (MCA-PSV) at 42.8 cm/s (1.6 multiple of median {MoM}; Figure 1). The patient underwent fetal blood sampling and IUT of 15 mL, and weekly assessment of MCA-PSV was accordingly done with multiple IUTs as illustrated in Table 1. At 26 weeks of gestation, after four episodes of IUT, the brain ultrasound showed bilateral severe ventriculomegaly, dilated third ventricle, and cerebellar cyst, likely representing periventricular leukomalacia due to brain hypoxia (Figure 2). The patient was counseled on the poor outcome of this rare fetal anemia complication and repeated IUT. The pregnancy continued until 34 weeks and five days, and the patient delivered a baby girl via an emergency C/S.

**Table 1:** Fetal MCA-PSV in relation to each intrauterine blood transfusion cycle MCA-PSV: middle cerebral artery-peak systolic velocity; MoM: multiple of median

Transfusion cycle no.	Gestational age	MCA-PSV (cm/s)	MCA-PSV (MoM)	Blood volume transfused (mL)
1	21 weeks and 5 days	42.8	1.6	15
2	24 weeks and 2 days	52.8	1.7	58
3	25 weeks and 4 days	62.5	1.9	44

Newborn findings are shown in Table 2. The newborn was admitted to the neonatal intensive care unit due to respiratory distress, anemia, and neonatal jaundice and was placed on continuous positive airway pressure. The newborn received O-negative blood transfusion at birth. Exchange transfusion and immunoglobulin were given 2.5 hours after birth. The newborn was started on intensive phototherapy. The serum bilirubin level decreased gradually to below the phototherapy zone after 40 hours, and the newborn was weaned gradually from oxygen to room air over a period of two days. The newborn had difficulty tolerating oral feeding, taking approximately two weeks to shift from tube to oral feeding.

**Table 2:** Newborn findings APGAR: appearance, pulse, grimace, activity, and respiration

Newborn findings	Values
APGAR score	7 and 8 at 1 and 10 min, respectively
Cord pH	Venous, 7.23 and arterial, 7.14
Weight	2080 g (falling on the 10th and 50th percentile)
Length	41 cm (third percentile)
Head circumference	32.5 cm (50th percentile)
Bilirubin	149 mmol/dL (jaundiced)
Hemoglobin	4.6 g/dL
Physical examination	Irritable, with high-pitched cry. Hypertonicity of the upper limbs. Spasticity of the lower limbs

**Figure 3:**
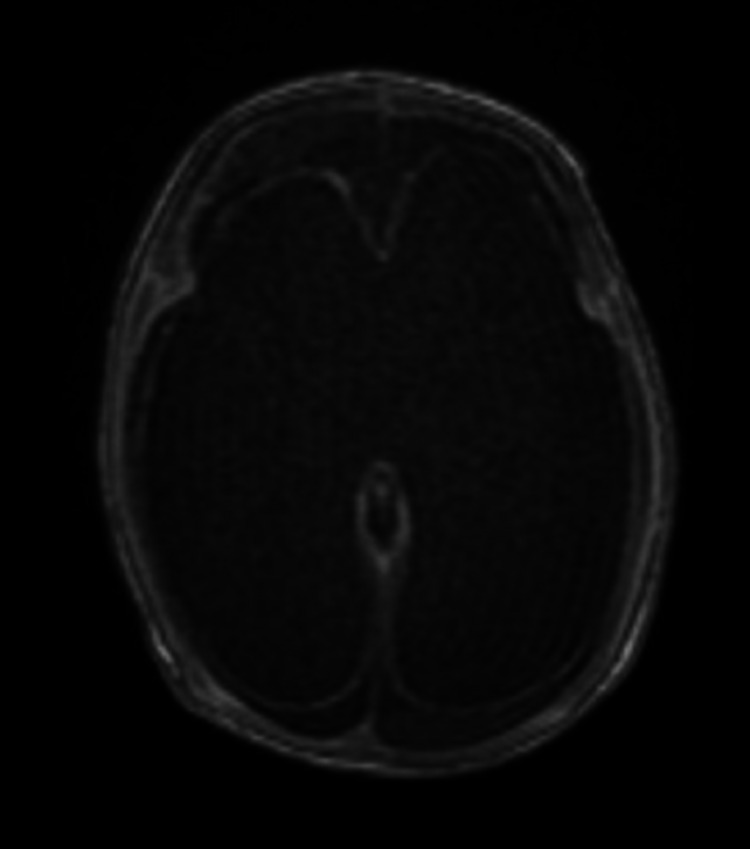
Postnatal brain magnetic resonance imaging of a patient with severe brain volume loss and mantel index thinning

Magnetic resonance imaging of the brain showed bilateral marked lateral ventricular dilatation, which appeared to be passive due to parenchymal volume loss with a very thin cortical mantel. The third and fourth ventricles were not dilated. No obstructive lesions were identified at the level of the foramen of Monro. Cystic encephalomalacia changes in the cerebellum were noticed (Figure 3). The newborn was discharged home with a follow-up appointment at the physiotherapy, neurology, and high-risk neonatal clinic in addition to follow-up at the clinic four weeks post discharge. The newborn showed bilateral optic nerve atrophy, hyperreflexia, and contracture on the lower limbs that needed surgical fixation; frequent choking on feeds; and findings of global developmental delay.

## Discussion

IUT is the gold standard of fetal anemia treatment, with a good prognosis and minor risk of fetal loss (1-3%) in uncomplicated procedures. However, the risk increases to about 20% in hydropic fetuses [1]. Fetal brain lesions can occur as complications of severe and prolonged intrauterine fetal anemia, leading to brain anoxia mainly due to delayed referral for intrauterine transfusion or technical difficulty. Dildy et al. reported a case of a porencephalic cyst related to a difficult intrauterine transfusion [2]. Brain lesions that are seen after IUT in severely anemic fetuses mimic sonographic neurological appearance similar to that observed in monochorionic twins after the demise of one, which supports the hypothesis that brain anomalies can develop after severe fetal anemia [3]. In addition, unilateral cerebellar hypoplasia was related to intrauterine ischemic or vascular injuries [4]. However, the patient in the current case developed encephalomalacia as a rare complication of fetal anemia due to Rh isoimmunization.

In a retrospective study, Pasman et al. have reported 135 IUTs in 56 fetuses, with minimal adverse effects of approximately 10%. They found that severe fetal effects occurred in only 1.5%, and hydrops and IUT in the free lope were a significant predisposing factor. However, IUT in the late gestation was associated with good fetal and neonatal outcomes [5].

The risk of anemia is considered high when MCA-PSV reaches ≥1.5 MoM, whereas levels below this value indicate that the fetus is not anemic or does not call for interventions [6]. MCA-PSV, which was normalized after transfusion, has been considered a standard for the detection of fetal anemia and the timing of IUT [7]. In few cases, it has been reported that IUT in the early second trimester was associated with higher fetal loss (6.7%) and increased probability of prenatal intracranial hemorrhage [8]. Despite successful IUT, fetuses with severe anemia were at increased risk of cerebral damage mainly between 24 and 32 weeks of gestation, in which the proliferation of neuroblast and differentiation of cerebral cortex take place [9].

The LOTUS study found that children (two to 17 years) who received IUT had normal neurodevelopment. This shows the safety of IUTs regardless of the number of procedures, with rare complications mainly related to premature delivery, hydrops fetuses, and low hemoglobin rather than the number of transfusions or procedures itself [10]. Severe anemia due to red blood cell alloimmunization seems to increase the risk of cerebellar damage when the fetal hemoglobin reaches a critical value (<2 g/dL). Moreover, hypoxic-ischemic encephalopathy takes seven to 14 days to be diagnosed by radiological modality [11].

## Conclusions

IUT is the gold standard for fetal anemia treatment. In cases of Rh isoimmunization, delayed treatment or complicated IUT procedures can cause fetal brain anoxia and brain lesions. Thus, timely and careful intervention by an experienced maternal fetal medicine specialist is crucial for superior fetal and neonatal outcomes.
